# Transcriptome Dynamics of *Brassica juncea* Leaves in Response to Omnivorous Beet Armyworm (*Spodoptera exigua*, Hübner)

**DOI:** 10.3390/ijms242316690

**Published:** 2023-11-24

**Authors:** Rui Xia, Liai Xu, Jiaojiao Hao, Lili Zhang, Shanyi Wang, Zhujun Zhu, Youjian Yu

**Affiliations:** Key Laboratory of Quality and Safety Control for Subtropical Fruit and Vegetable, Ministry of Agriculture and Rural Affairs, Collaborative Innovation Center for Efficient and Green Production of Agriculture in Mountainous Areas of Zhejiang Province, College of Horticulture Science, Zhejiang A&F University, Hangzhou 311300, China; 2020101032021@stu.zafu.edu.cn (R.X.); 20210035@zafu.edu.cn (L.X.); 2021114012020@stu.zafu.edu.cn (J.H.); 2021114012016@stu.zafu.edu.cn (L.Z.); 2021614022033@stu.zafu.edu.cn (S.W.)

**Keywords:** mustard, herbivory, glucosinolate, sinigrin, jasmonic acid, insect resistance

## Abstract

Cruciferous plants manufacture glucosinolates (GSLs) as special and important defense compounds against insects. However, how insect feeding induces glucosinolates in Brassica to mediate insect resistance, and how plants regulate the strength of anti-insect defense response during insect feeding, remains unclear. Here, mustard (*Brassica juncea*), a widely cultivated Brassica plant, and beet armyworm (*Spodoptera exigua*), an economically important polyphagous pest of many crops, were used to analyze the changes in GSLs and transcriptome of Brassica during insect feeding, thereby revealing the plant–insect interaction in Brassica plants. The results showed that the content of GSLs began to significantly increase after 48 h of herbivory by *S. exigua*, with sinigrin as the main component. Transcriptome analysis showed that a total of 8940 DEGs were identified in mustard challenged with beet armyworm larvae. The functional enrichment results revealed that the pathways related to the biosynthesis of glucosinolate and jasmonic acid were significantly enriched by upregulated DEGs, suggesting that mustard might provide a defense against herbivory by inducing JA biosynthesis and then promoting GSL accumulation. Surprisingly, genes regulating JA catabolism and inactivation were also activated, and both JA signaling repressors (JAZs and JAMs) and activators (MYCs and NACs) were upregulated during herbivory. Taken together, our results indicate that the accumulation of GSLs regulated by JA signaling, and the regulation of active and inactive JA compound conversion, as well as the activation of JA signaling repressors and activators, collectively control the anti-insect defense response and avoid over-stunted growth in mustard during insect feeding.

## 1. Introduction

Plants are exposed to highly complex and variable environmental conditions during their growth and development, and are susceptible to a variety of biotic and abiotic stress conditions. Once plants are attacked by insects, a series of effective defense mechanisms are activated [1,2], including mechanical defenses and chemical defenses, which are the two main types of plant defenses. When plants are invaded by insects, defense signals spread from attack sites and generate self-defense responses in distal regions of the same plant, thus triggering a systemic defense [3]. Moreover, the insect-induced plant defense response is mainly achieved by activating the expression of defense genes, thereby generating and activating defense enzyme activity, and synthesizing defensive substances [4].

Phytohormones are important regulators involved in the perception and transmission of various environmental signals and subsequent defense responses [5]. In general, insect-induced defense responses are mainly mediated by jasmonic acid (JA), salicylic acid (SA), and ethylene (ET), and these hormone-related pathways interact in complex ways at the transcriptional levels [6,7]. JA is widely present in higher plants and plays an important role in the regulation of plant defense-related genes [5,8]. When plants are damaged by herbivores, linoleic acid is released from plant cell membrane lipids and converted into active jasmonic acid through the octadecanoid pathway mediated by various enzymes. Subsequently, the JA signaling transduction pathway is triggered, and the synthesized JA binds to membrane receptors, initiating transcription of defense genes, and activating other JA-mediated responses [9]. As early as 2007, studies showed that both mechanical injury and insect feeding can lead to rapid and transient accumulation of JA at the injured site, thus activating the expression of defense genes around the wound and generating local defense responses [10]. Even at present, the field of JA-related plant stress responses and defense mechanisms still receives extensive attention.

The defensive chemicals produced by plants are important executors of plant chemical defenses, aiding plants in escaping herbivory. Most of those chemicals used for defensive purposes are secondary metabolites derived from primary metabolites, typically including terpenes, phenolics and nitrogen compounds [11,12,13]. Notably, many secondary metabolites with a chemical defense function can be induced by JA [14,15]. Among these defensive chemicals, glucosinolates (GSLs) are hydrophilic secondary metabolites containing sulfur and nitrogen [16], mainly present in cruciferous plants such as mustard, cabbage, and *Arabidopsis thaliana* [17]. Evidence has shown that the resistance of mustard (*Brassica juncea*) to insects was positively correlated with the accumulation of GSLs in the plants [18,19]. GSLs can be classified into aliphatic, indolic, and aromatic GSLs according to their amino acid side-chain R groups [20]. Various modified GSLs are generated after their amino acid side chains are altered through different processes, including hydroxylation, O-methylation, alkenylation, desaturation or benzoylation [16,21]. To date, the biosynthesis of GSLs and their regulation in plants has been intensively and well studied [20,22]. 

Transcription factors (TFs) are widely involved in various biological processes and are of considerable significance in the gene expression regulation response to biotic stress [23]. It has been reported that MYB and MYC TFs are two major regulators of GSL biosynthesis [24,25]. *MYB28*, *MYB29* and *MYB76* are mainly involved in regulating the biosynthesis of aliphatic GSLs [26,27], while *MYB34*, *MYB51*, and *MYB122* mainly enhance the biosynthesis of indolic GSLs [28]. *MYC2*, *MYC3*, and *MYC4* belong to the family of MYC TFs which interact with MYBs and directly regulate GSL biosynthesis [20,25]. It is noteworthy to mention that the metabolism and regulation of GSLs are also controlled by phytohormones, especially JA [20]. It has been demonstrated that the regulation of GSL biosynthesis by JA depends on the COI1/JAZ/MYC2 pathway mediated by jasminoyl-L-isoleucine (JA-Ile). When the endogenous JA level is lower than the threshold concentration, the expression of JA-response genes is switched off by inhibiting the transcription of MYC TFs [29]. However, how do plants defend themselves against insects by affecting MYC expression and GSL accumulation through JA signaling? This issue still needs further exploration. Moreover, existing research on plant–insect interactions mostly focuses on the possible mechanisms by which plants defend themselves against insect feeding, with few studies discussing how plants regulate the strength of anti-insect defense response to avoid over-stunted growth due to excess defense response when subjected to insect stress.

*Brassica juncea* is an economically important Brassica species with multiple genotypes and is widely distributed around the world, including vegetable mustard [30,31], which is extensively cultivated in China. However, the damage of pests and diseases seriously affects the yield and economic benefits of mustard. Beet armyworm (*Spodoptera exigua*, Hubner) is a polyphagous pest widely distributed around the world and is an economically important pest of many crops, including mustard [32]. Remarkably, an aliphatic GSL, sinigrin (SIN), is the main GSL in mustard, accounting for approximately 95% of the total GSLs in its leaves [33]. The genetic and genomic information of the vegetable mustard have already been published through whole-genome sequencing [31]. However, with SIN as the predominant GSL, comparative investigation of the GSL profile and expression patterns of genes responding to insect chewing on leaves of vegetable mustard are still limited. Therefore, it is of great significance for pest control to explore the relationship between GSL metabolism and insect pests.

In this study, we investigated the changes in the GSL profile and transcriptome of mustard leaves when challenged with omnivorous pest beet armyworm larvae. Meanwhile, we also focus on the possible molecular mechanisms by which Brassica plants regulate the strength of anti-insect defenses response during herbivory from transcriptome data. Our results provide valuable information for further clarifying the molecular regulatory mechanism of Brassica plant/herbivory interplay and are also beneficial for breeding insect-resistant Brassica plants.

## 2. Results

### 2.1. Changes in Glucosinolate Profiles in Mustard Leaves after Beet Armyworm Larvae Chewing

**Figure 1:**
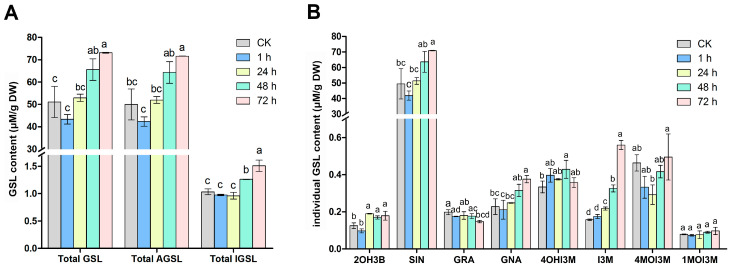
Changes in GSL constituents and contents in the leaves of *Brassica juncea* after challenge with beet armyworm larvae for 0, 1, 24, 48 and 72 h. (**A**) Total GSLs, aliphatic GSLs and indolic GSLs. (**B**) Individual GSLs. Data are the mean ± standard error from three biological replicate assays (*n* = 3). Different letters show significant differences (*p* < 0.05) for each sampling data among treatments. DW, dry weight; GSL, glucosinolate; AGSL, aliphatic GSL; IGSL, indolic GSL; 2OH3B, progoitrin; SIN, sinigrin; GRA, glucoraphanin; GNA, gluconapin; 4OHI3M, 4-OH-glucobrassicin; I3M, glucobrassicin; 4MOI3M, 4-methoxy-glucobrassicin; 1MOI3M, 1-methoxy-glucobrassicin.

To investigate how insect chewing affects glucosinolate metabolism, we first determined the GSL profiles of mustard leaves after challenge with beet armyworm larvae for 0, 1, 24, 48 and 72 h. Total GSLs did not change after 1 and 24 h of challenge, but increased significantly after 48 and 72 h (Figure 1A). A total of eight GSLs were identified in mustard leaves, including four aliphatic GSLs (2OH3B, SIN, GRA, GNA) and four indolic GSLs (4OHI3M, I3M, 4MOI3M, 1MOI3M), but no aromatic GSLs were detected. Aliphatic GSLs were the major GSLs, accounting for approximately 98% of the total GSLs, whereas indolic GSLs only account for 2%. Overall, the content of aliphatic and indolic GSLs increased gradually with the chewing time of beet armyworm larvae, which increased by 30.1% and 31.6%, respectively, compared with the control after 72 h of chewing (Figure 1B, Table S1). Sinigrin (SIN), an aliphatic GSL accounting for about 97% of the total GSLs, increased by 30.2% after 72 h of challenge. In addition, the other two aliphatic GSLs, progoitrin (2OH3B) and gluconapin (GNA), also began to increase significantly after 24 and 72 h of challenge, respectively. However, the content of glucoraphanin (GRA) did not change significantly. For indolic GSLs, we found that the contents of 4OHI3M and 1MOI3M were relatively stable, while I3M began to increase significantly at 24 h and reached the maximum at 72 h. Interestingly, the content of 4MOI3M appeared to decrease at 24 h and then recovered to the level before insect chewing. However, no new GSLs were detected during beet armyworm larvae chewing (Figure 1, Table S1).

### 2.2. Transcriptome Sequencing Analysis

In order to investigate the expression changes in transcripts in mustard leaves during the feeding process of the beet armyworm larvae, the transcriptomes of mustard leaves challenged with beet armyworm larvae for 0, 1, 24, 48 and 72 h were analyzed using the Illumina HiSeq high-throughput sequencing platform. A summary of the sequencing data is shown in Table S2. After sequencing quality control, a total of 66.36 G clean bases were obtained from 10 cDNA libraries, and each sample reached 5.8 G. Moreover, the percentages of Q30 bases were all over 90%, and the GC content of each sample was about 48%. The clean reads of each sample were aligned with the reference genome of *B. juncea*, and the alignment efficiency ranged from 90.29% to 91.77%. The alignment results were further used for reference and guidance for subsequent assembly and differential gene expression analysis. FPKM values were used to measure the gene expression levels of mustard at different time points during insect feeding. The results showed that the overall gene expression levels of different samples exhibited similar expression profiles, indicating that the collection and analysis methods of biological samples were reliable (Figure S1).

### 2.3. Dynamic Expression Pattern of DEGs 

**Figure 2:**
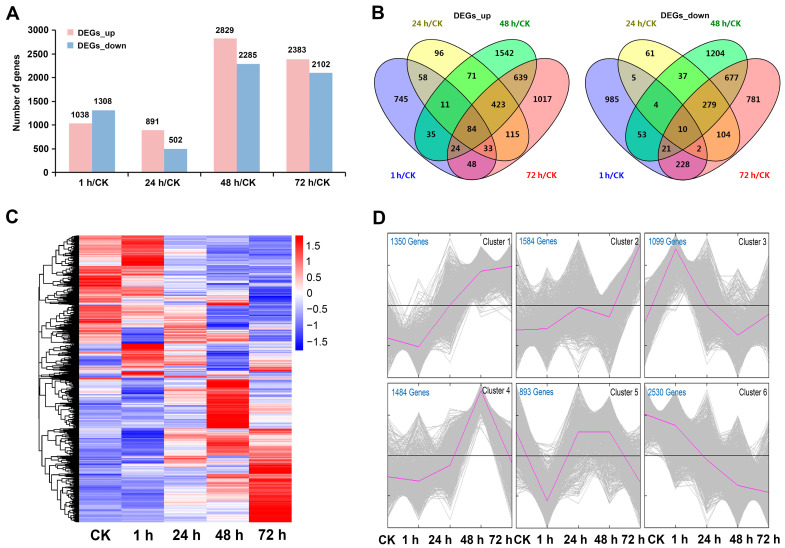
DEGs analysis of mustard leaves infested with beet armyworm larvae for 0 (CK), 1, 24, 48 and 72 h. (**A**) The numbers of up- and downregulated DEGs at four time points compared to unchallenged leaves. (**B**) Venn diagram showing the number of specific and shared up- or downregulated DEGs between the four time points. (**C**) Hierarchical clustering heatmap showing the expression patterns of DEGs after beet armyworm larvae chewing. (**D**) Time-course expression patterns of DEGs. X-axis represents the five insect chewing time points of *Brassica juncea* (CK, 1, 24, 48 and 72 h). Y-axis represents the normalized value of the DEGs expression level.

To analyze the differences in transcription levels of mustard leaves during insect pest stress periods, differentially expressed genes (DEGs, *q*-value ≤ 0.05 and |log2 (foldchange)| ≥ 1) at different time points after beet armyworm larvae chewing were identified. A total of 8940 DEGs were identified in at least one pairwise comparison between the four time points after beet armyworm larvae chewing and the control. In each comparison, except for 1 h/CK, the number of upregulated DEGs was higher than that of downregulated DEGs. Overall, more DEGs were identified in 48 h/CK and 72 h/CK, but less in 1 h/CK and 24 h/CK (Figure 2A, Table S3). The total number of DEGs in the four groups was 48 h/CK, 72 h/CK, 1 h/CK, 24 h/CK in descending order. Similarly, the Venn diagram showed that the number of specific up- and downregulated DEGs in each group also had the same order. As expected, 48 h/CK and 72 h/CK shared the most DEGs in both up- and downregulated DEGs (Figure 2B, Table S4). In conclusion, the transcriptome of mustard leaves showed relatively mild changes at 1 h and 24 h of beet armyworm larvae chewing, but there were significant changes at 48 h and 72 h. 

In order to clearly represent the relationship between the transcriptome changes of mustard leaves and chewing time, hierarchical clustering was used to display the expression of 8940 DEGs over time. The heatmap showed that these DEGs had different expression patterns at different time points. The difference of the DEGs expression patterns between 48 h and 72 h after challenge with beet armyworm larvae was greater than that at 1 h and 24 h (Figure 2C). To further demonstrate the time-course of DEGs expression in mustard leaves during herbivory, all DEGs were grouped into six clusters based on their temporal expression. Cluster 1 contained 1350 DEGs that were gradually upregulated with the extension in insect feeding time. The expression of 1584 DEGs in Cluster 2 did not change much in the pre- and mid-infestation, but reached the highest at 72 h. The 1099 DEGs of Cluster 3 and the 1484 DEGs of Cluster 4 achieved maximum expression at 1 h and 48 h, respectively. Cluster 5 contained the least number of DEGs, showing decreased expression at 1 h and 72 h, but there seemed to be no significant difference between plants challenged with insects for 24 h and 48 h and those unchallenged. In contrast to Cluster 1, the expression of the 2530 DEGs in Cluster 6 gradually decreased with the prolongation of insect feeding time (Figure 2D, Table S5).

### 2.4. Functional Analysis of DEGs

**Figure 3:**
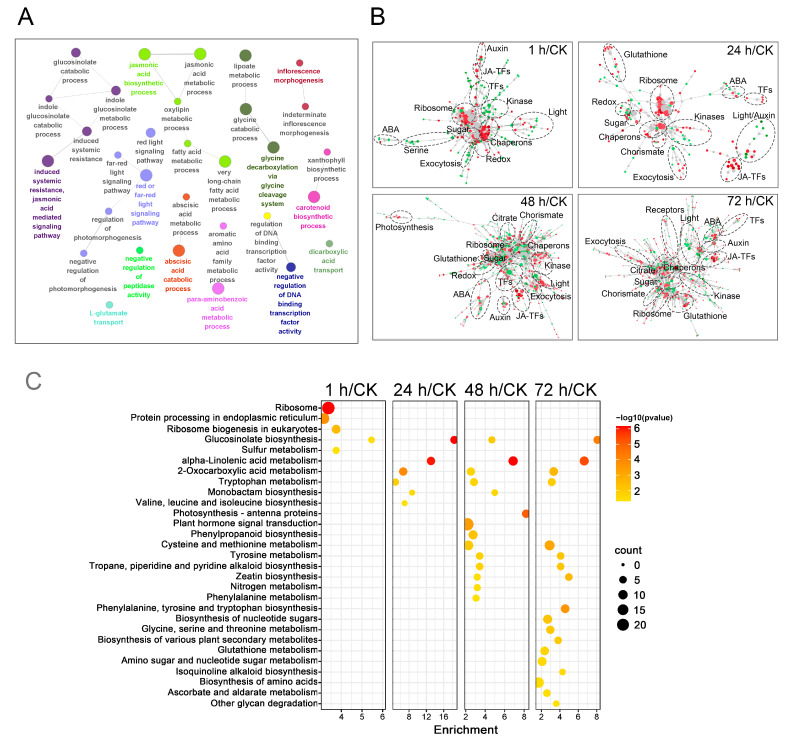
Classification of DEGs in mustard leaves chewed by beet armyworm larvae according to GO database, minimum connected molecular networks and KEGG database. (**A**) GO enrichment analysis of DEGs. (**B**) Minimum connected molecular networks based on DEGs in each time point and the PPI from STRING database. Red points, upregulated DEGs; green points, downregulated DEGs; gray points, non-seed DEGs. (**C**) KEGG classification of upregulated DEGs. The X-axis represents the enrichment, which is the ratio of the number of DEGs to the total number of genes in a certain pathway. The color and size of the dots represent the range of −log10 (*p*-value) and the number of DEGs mapped to the indicated pathways, respectively.

To explore which biological processes are affected in mustard leaves over time after beet armyworm larvae chewing, GO analysis was carried out on all DEGs identified above. The results demonstrated that several GO terms related to the jasmonic acid metabolic process, glucosinolate metabolic process, abscisic acid catabolic process and glycine catabolic process were significantly enriched (Figure 3A). Gene network analysis revealed that those gene modules associated with hormonal responses (JA, ABA and Auxin), exocytosis enhancement, ribosome, chaperons, sugar and glutathione (Figure 3B). DEGs were further used in the KEGG database to analyze metabolic pathways. The results showed that there were similarities and differences in the KEGG pathways that were significantly enriched by upregulated DEGs at four time points after insect feeding (Figure 3C, Table S6). The glucosinolate biosynthesis pathway was the only KEGG pathway that was significantly enriched at all four time points. The alpha-linolenic acid metabolism, 2-Oxocarboxylic acid metabolism and tryptophan metabolism pathways were all enriched at the last three time points. Notably, the latter two time points had more common significantly enriched pathways for upregulated DEGs. These results suggested that the upregulated DEGs of mustard after challenge with beet armyworm larvae were mainly related to jasmonic acid metabolic process as well as glucosinolate biosynthesis and the metabolic process. By contrast, downregulated DEGs did not present any particular pattern (Figure S2, Table S7).

### 2.5. Expression Patterns of Genes Involved in Glucosinolate Biosynthesis

**Figure 4:**
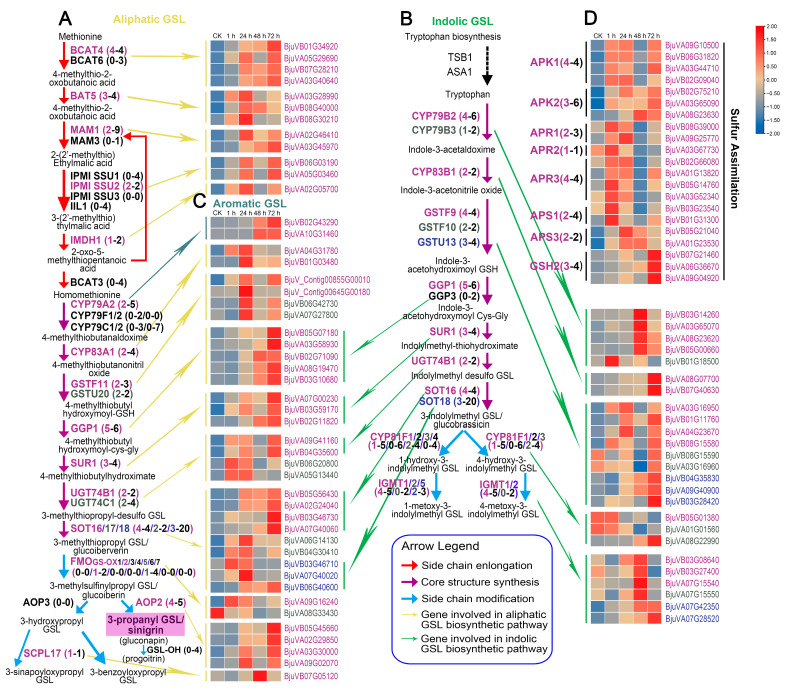
Biosynthetic pathways of aliphatic and indolic glucosinolates (GSLs) and the heatmap of related *BjuGSLs* responding to beet armyworm larvae chewing in mustard. The biosynthetic pathway of aliphatic (**A**), indolic (**B**), aromatic (**C**) GSLs, and sulfur assimilation (**D**) and the heatmap of related *BjuGSLs*. The scale bar represents FPKM values. The number in parentheses represents the copy number of the key genes related to GSL biosynthesis; the first one means the corresponding DEGs; the second one means total copy of the gene. Abbreviations: BCAT, branched-chain amino acid aminotransferase; BAT5, probable sodium/metabolite cotransporter BASS5; MAM, methylthioalkylmalate synthase; IPMI-SSU, isopropylmalate isomerase small subunit; IIL1, isopropylmalate isomerase large subunit 1; IMDH1, isopropylmalate dehydrogenase 1; CYP79A, phenylalanine N-monooxygenase; CYP79B, tryptophan N-monooxygenase; CYP79C/F, cytochrome P450 79C/F; CYP83B, CYP83B monooxygenase; GSTF/U, glutathione S-transferase F/U; GGP1, γ-glutamyl peptidase 1; SUR1, C-S lyase 1; SOT, sulfotransferase; FMOGS-OX, flavin-containing monooxygenase; AOP2, 2-oxoglutarate-dependent dioxygenase; SCPL17, serine carboxypeptidase-like 17; IGMT, indole GSL O-methyltransferase; APK, adenylyl-sulfate kinase; APR, 5′-adenylylsulfate reductase; APS, ATP sulfurylase; GSH2, glutathione synthetase. The framework of the aliphatic, indolic biosynthetic pathways and sulfur assimilation is adapted from ref. [34].

GO and KEGG enrichment analysis indicated that GSL biosynthesis was involved in the plant response to beet armyworm larvae chewing. For more details, the expression of GSL biosynthesis-related genes (*BjuGSLs*) at different time points after herbivory was further analyzed. Based on the latest whole-genome and transcriptome data of vegetable mustard, we identified a total of 209 *BjuGSLs* (including sulfur assimilation-related genes) by comparing their related homologs in *A. thaliana*. Among them, 107 were derived from the *B. juncea* A subgenome, 98 from the *B. juncea* B subgenome, and there were also 4 new genes (Figure 4, Table S8). Among them, 94 were differentially expressed at least at one time point after herbivory (Figure 4, Table S8). Remarkably, of the 94 DEGs, 84 were upregulated at least at one time point, while only 16 were downregulated, of which 6 were up- and downregulated at different time points (Figure S3). The upregulation of a large number of GSL DEGs was consistent with the phenotype of elevated total GSL content, which again proved that GSL synthesis was closely related to insect resistance.

Among the ten proteins involved in aliphatic GSL chain elongation, 4, 3, 2, 2 and 1 DEGs encoding *BCAT4*, *BAT5*, *MAM1*, *IPMI SSU2* and *IMDH1*, respectively, were upregulated after insect feeding, while the expression of 16 genes encoding the other five proteins did not change significantly (Figure 4A). There were 20 proteins involved in the formation of the core structure of aliphatic and/or indolic GSLs. Among them, eight proteins were only responsible for the synthesis of aliphatic GSLs, involving 27 genes, 10 of which were DEGs. Seven proteins encoded by 22 genes were only responsible for the core structure formation of indolic GSLs, and as many as sixteen of them were DEGs (Figure 4B). Five of the twenty proteins were involved in the synthesis of both aliphatic and indolic GSLs, and were encoded by 36 genes, including 20 *SOT18* genes. Notably, despite only 17 DEGs out of 36 genes, four DEGs encoding *SOT16* were significantly upregulated and highly expressed after challenge with beet armyworm larvae (Figure 4A,B). In addition, for genes related to the synthesis of aromatic GSL core structure, two *CYP79A2* DEGs were identified, which were upregulated when challenged for 48 h (Figure 4C). 

The enzymatic systems responsible for side-chain modification of aliphatic GSLs and indolic GSLs are different. For aliphatic GSLs, the side-chain modification is initiated by flavin monooxygenases (FMO_GS-OXs_), followed by side-chain oxygenation catalyzed by 2-oxoglutarate-dependent dioxygenases (AOP2 and AOP3), and finally by GSL-OH or SCP17 to further convert alkenyl GSLs to hydroxylated alkenyl GSLs, or hydroxyalkyl GSLs to benzoylated GSLs (BzGSLs) and sinapoylated GSLs (SnGSLs). Here, we found that there are seven *FMO_GS-OXs_* (*FMO_GS-OX1-7_*) in *Arabidopsis*, but only two, *FMO_GS-OX2_* and *FMO_GS-OX5_*, are retained in mustard, which are encoded by two and four paralogous genes, respectively. Transcriptome data showed that only one of these six genes was significantly upregulated after insect feeding. *AOP2*, *GSL-OH* and *SCPL17* are still retained in mustard, encoded by 5, 4 and 1 genes, respectively, but *AOP3* has been lost. It should be noted that the four out of five genes encoding *AOP2* were significantly upregulated after insect feeding, while none of the four genes encoding *GSL-OH* were upregulated (Figure 4A). This was consistent with the result that mustard were highly enriched in alkenylated aliphatic GSL named sinigrin (Figure 1B). For indolic GSLs, the cytochrome P450 monooxygenases of the *CYP81F* subfamily (*CYP81F1*-*4*) and the indole glucosinolate O-methyltransferases (*IGMTs*, *IGMT1*/*2*/*5*) are responsible for the side-chain modification (Figure 4B). Although there were multiple paralogous genes encoding each of these proteins in mustard, their basal expression was generally low, and most genes did not show differential expression after insect feeding, especially the genes encoding *CYP81Fs*. These findings also partly explain why the side-chain-modified indolic GSL (4OHI3M, 4MOI3M and 1MOI3M) did not increase significantly after insect feeding (Figure 4B). 

The sulfur assimilation process is expected to greatly influence GSL biosynthesis as the core structure of GSLs usually contains at least two sulfur atoms. Ten proteins were identified to be involved in the sulfur assimilation process, including two adenylyl-sulfate kinases (APK1/2), three 5′-adenylylsulfate reductases (APR1/2/3), two ATP-sulfurylases (APS1/3), one cysteine synthase (OASA1), one glutamate-cysteine ligase (GSH1) and one glutathione synthetase (GSH2) (Figure 4D, Table S8). A total of 37 genes encoded these ten proteins in mustard, of which 15 were upregulated DEGs. Interestingly, many DEGs were upregulated at 1 h, 24 h, and 72 h of insect feeding, but decreased to pre-insect feeding levels at 48 h. In general, the upregulation of these DEGs mostly started at 1 h after insect feeding, which was earlier than the DEGs involved in the three main processes of GSL biosynthesis. This difference indicated that the response of sulfur assimilation pathway to beet armyworm larvae chewing was earlier than that of GSL biosynthesis process. 

**Figure 5:**
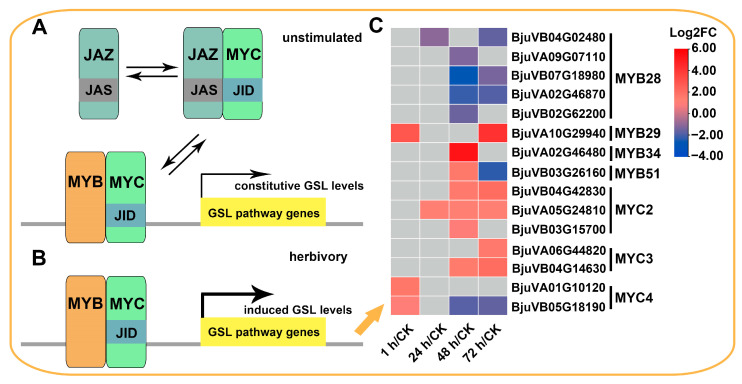
Regulation of GSL biosynthesis by MYB and MYC transcription factors of beet armyworm larvae defense responses. (**A**) When unstimulated, the JAZ inhibitor binds to the JID domain of the MYC transcription factor and thus inhibits MYC/MYB interaction. (**B**) When a plant is attacked by insects and activates the JA pathway, the JAZ suppressor is removed from the JID domain, thereby enhancing the interaction between MYBs and MYCs and thus enhancing the expression of genes related to the GSL biosynthesis pathway. (**C**) Heatmap showing the expression patterns of MYC and MYB DEGs after beet armyworm larvae chewing. Expression data are plotted as log2FC. The framework of the pathway is adapted from ref. [25].

The complex formed by MYC and MYB TFs is required for the direct transcriptional regulation of GSL biosynthesis. In mustard, 4, 4, and 2 paralogs were identified to encode *MYC2*/*3*/*4*, respectively, of which 3, 2, and 2 were DEGs (Figure 5, Table S9). Among these DEGs, three *BjuMYC2* and two *BjuMYC3* genes were upregulated after challenged with beet armyworm larvae for 48 h and 72 h. However, two *BjuMYC4* DEGs were upregulated at 1 h, with one of them was downregulated at 48 h and 72 h. Three *MYBs* (*MYB28*, *MYB29*, and *MYB76*) are central to aliphatic and three (*MYB34*, *MY51*, and *MYB122*) to indolic GSL biosynthesis. A total of 26 genes have been identified to encode these *MYBs* in mustard, of which *MYB76* is absent. Interestingly, only a *BjuMYB29* (*BjuVA10G29940*), a *BjuMYB34* (*BjuVA02G46480*), and a *BjuMYB51* (*BjuVB03G26160*) were up or downregulated in response to beet armyworm larvae. Moreover, five out of six *BjuMYB28* genes were downregulated. These findings indicated that the upregulation of *BjuGSLs* mentioned above may be more closely related to the upregulation of *BjuMYCs*, and as for *BjuMYBs*, the upregulation of a copy gene (*BjuVA10G29940*) of *BjuMYB29s* may also contribute significantly.

### 2.6. Multiple JA Biosynthesis-Related Genes in Mustard Were Upregulated after Insect Feeding

**Figure 6:**
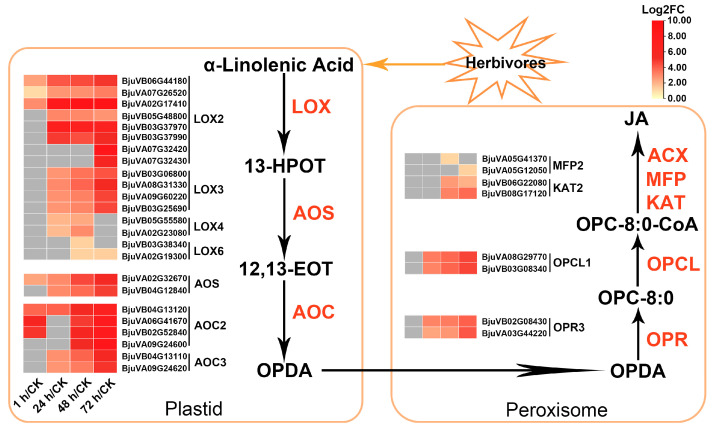
DEGs expression pattern diagram of JA biosynthetic pathway in mustard leaves after beet armyworm larvae chewing. Expression data are plotted as log2FC values. LOX, lipoxygenase; AOS, allene oxide synthase; AOC, allene oxide cyclase; OPR3, OPDA reductase 3; OPCL1, OPC-8:0-CoA ligase 1; KAT, 3-ketoacyl-CoA thiolase; MFP, multifunctional protein; ACX, acyl-CoA oxidase.

As the precursor of JA synthesis, the metabolism of alpha-linolenic acid has a great impact on the biosynthesis of JA. The above KEGG enrichment analysis results showed that the “alpha-Linolenic acid metabolism” pathway was significantly enriched at the last three time points, indicating that JA biosynthesis-related genes may be significantly induced at those time points. To further elucidate the dynamic changes of these genes at various time points after herbivory, the genes involved in the JA biosynthesis pathway in transcriptome data were analyzed. In mustard, a total of 54 genes encoding enzymes were identified, involved in six steps of JA biosynthesis starting from alpha-linolenic acid (Table S10). The first three steps are carried out in plastids, which catalyze the synthesis of 12-oxo-phytodienoic acid (OPDA) from alpha-linolenic acid and involve three types of enzymes, including lipoxygenases (LOXs), allene oxide synthases (AOSs), and allene oxide cyclases (AOCs). Notably, 24 out of the 32 genes encoding these three types of enzymes were upregulated on at least one time point after insect feeding, but none were downregulated (Figure 6, Table S10). Specifically, a total of 21 genes encoding LOXs were identified in mustard, with 11/4/2/4 genes encoding *LOX2*/*3*/*4*/*6*, respectively (Table S10). Among them, 16 were upregulated DEGs, most of which showed significant upregulation after 1 or 24 h of insect feeding and persisted until 72 h, especially the three DEGs (*BjuVA02G17410*, *BjuVB06G44180* and *BjuVA07G26520*) encoding LOX2s (Figure 6 and Table S10). The other two enzymes, AOS and AOC, are encoded by two and nine paralogs, respectively. Except for one *AOC3* and two *AOC4* genes, the remaining genes were upregulated DEGs. The OPDA synthesized in the plastid is then transferred to the peroxisome, and JA is synthesized after three enzyme catalytic steps, involving five types of enzymes. Among them, OPR3 and OPC-8:0-CoA ligase (OPCL1) are responsible for catalyzing OPDA to synthesize OPC-8:0 and OPC-8:0-CoA in turn, both of which are encoded by two genes, and these four genes begin to be significantly upregulated after 24 h of herbivory. The three other types of enzymes are involved in β-oxidation of OPC-8:0-CoA to JA, including ACX, MFP and KAT, which are encoded by 18 genes in mustard, of which 4 genes (1 *MFP* gene and 3 *KAT* genes) were upregulated for at least one time point after herbivory. Interestingly, as a precursor of JA, the anabolism of alpha-linolenic acid may not be more active, as its synthesis-related genes were not significantly upregulated, and several genes (e.g., *FAD7*/*8*) were even downregulated after beet armyworm larvae chewing (Table S10). In addition, only a few genes involved in the biosynthesis of other hormones (including auxin, abscisic acid, ethylene and salicylic acid) were found to be differentially expressed after insect feeding (Table S11). These findings suggest that the feeding by beet armyworm larvae may mainly induce the biosynthesis of JA, but has no significant impact on the metabolism of other hormones. Moreover, this induction effect mainly starts from promoting the conversion of alpha-linolenic acid to JA synthesis, rather than from promoting the biosynthesis of linolenic acid.

### 2.7. Genes Related to JA Metabolism and Repression of JA Signaling Were Significantly Induced after Insect Feeding

Previous studies have shown that genes encoding enzymes in GSL biosynthesis are JA-inducible, and the concentration of GSLs is partially controlled by the JA pathway [35]. Therefore, the upregulation of JA and GSL biosynthesis genes and the accumulation of GSLs shown in the above results can be partially explained by insect-feeding-induced JA synthesis, which in turn activates the expression of *BjuGSLs* and promotes GSL synthesis. In addition, the JA-induced expression of multiple marker genes (e.g., mustard homologous genes of *VSP1*/*2* and *JR2*) of the wound response was significantly activated immediately after insect feeding (Table S13). So, will the anti-herbivory defense response induced by JA remain activated or will it be regulated to avoid over-stunted growth due to excess defense response? To explore this issue, we further analyzed the expression profiles of JA metabolism and JA signaling-related genes.

#### 2.7.1. JA Metabolism

**Figure 7:**
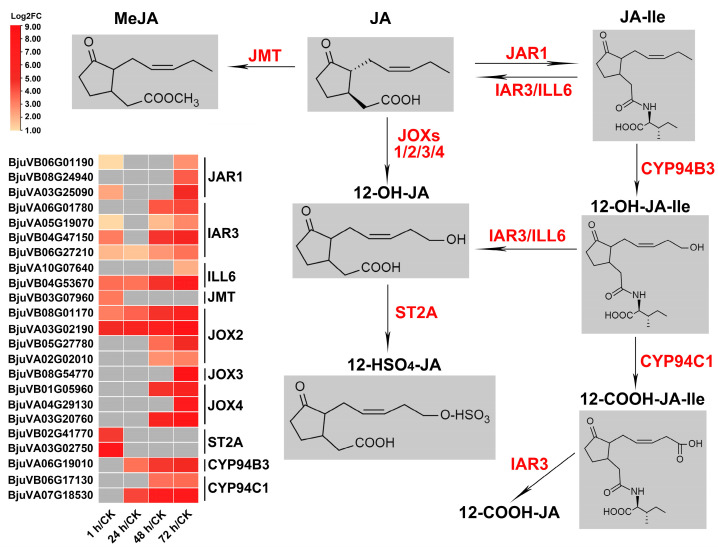
DEGs expression pattern diagram of JA metabolism responding to insect herbivores in mustard leaves. Expression data are plotted as log2FC values. JAR1, jasmonoyl isoleucine synthetase 1; IAR3, jasmonic acid responsive 3; ILL6, IAA-leucine resistant (ILR)-like gene 6; JMT, JA methyl transferase; JOX, jasmonate-induced oxygenases; ST2A, 12-OH-JA sulfotransferase; CYP94B3, JA-Ile-12-hydroxylase; CYP94C1, 12-OH-JA-Ile carboxylase.

JA-Ile has been identified as the most biologically active JA compound, formed by jasmonoyl-isoleucine synthetase (JAR1) catalyzing the conjugation between JA and isoleucine. In mustard, there are a total of six paralogs encoding JAR1, and as expected, three genes (*BjuVB06G01190*, *BjuVB08G24940*, *BjuVA03G25090*) were significantly upregulated for at least one time point after herbivory, which is consistent with the induction of JA synthesis and JA response after insect feeding (Figure 7, Table S12). Hydroxylation of JA-Ile to 12-OH-JA-Ile is one of the metabolic pathways that converts JA into inactive compounds, which is catalyzed by CYP94B3. 12-OH-JA-Ile can also be further carboxylated to 12-COOH-JA-Ile by CYP94C1. Interestingly, one of the four mustard genes (*BjuVA06G19010*) encoding CYP94B3 and both genes (*BjuVB06G17130*, *BjuVA07G18530*) encoding CYP94C1 were significantly upregulated after 24 or 48 h of herbivory, and persisted until 72 h. The amido-hydrolases IAR3 and ILL6 are responsible for cleaving JA-Ile and 12-OH-JA-Ile into JA and 12-OH-JA, and IAR3 may also catalyze the formation of 12-COOH-JA-Ile into 12-COOH-JA. Transcriptome data showed that all six mustard homologous genes encoding these two enzymes were significantly induced after insect feeding, and most of them began to be activated 1 h after feeding (Table S12). JA methyl transferase (JMT) and jasmonate-induced oxygenases (JOXs) directly methylated and esterified or hydroxylated JA to form MeJA and 12-OH-JA, respectively. Only one of the six genes encoding JMT was upregulated after 1 h of insect feeding, but half of the mustard JOXs encoding genes (eight out of sixteen) were upregulated at multiple time points. In addition, there was also a mustard gene encoding ST2a upregulated after 1 h of insect feeding, which was responsible for sulfation of 12-OH-JA. In general, although *JAR1*, which converts JA into active JA-Ile, was upregulated after insect feeding, more genes regulating JA-Ile catabolism and inactivation were activated (Figure 7, Table S12), indicating that the homeostasis of JA-Ile was strictly and complexly regulated during beet armyworm larvae chewing.

#### 2.7.2. JA Signaling

**Figure 8:**
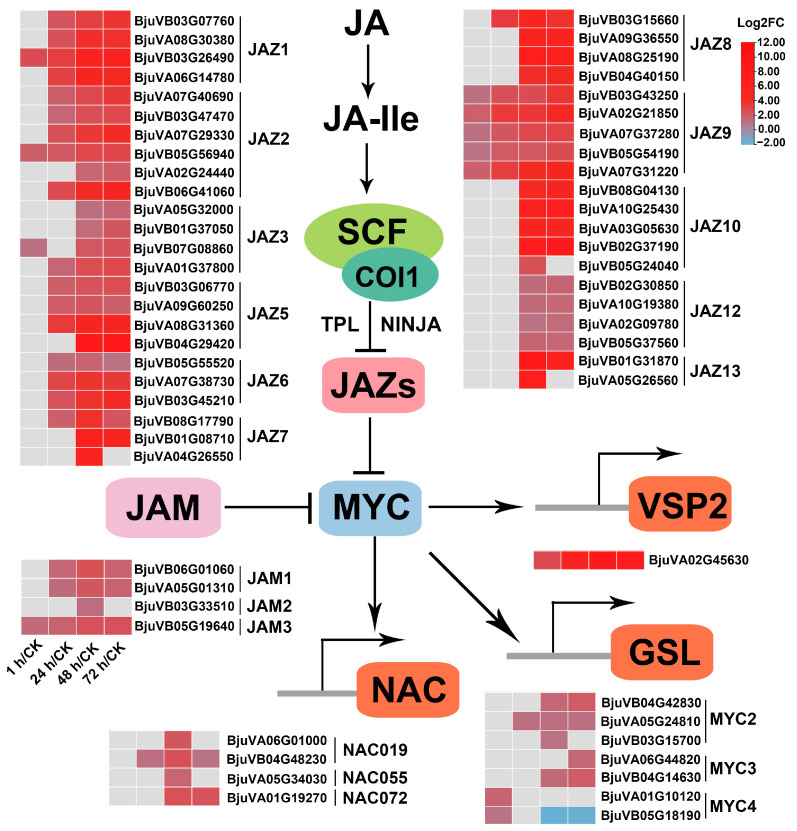
Heatmap of differentially expressed JA signaling-related genes in mustard leaves after beet armyworm larvae chewing. Expression data are plotted as log2FC values. SCF, [kinetochore protein 1 (SKP1)-CULLIN1 (CUL1)-F-box]; COI1, coronatine insensitive1; JAZ, jasmonate ZIM domain; JAM, jasmonate-associated MYC2-like TF; VSP2, vegetative storage protein 2; NAC, NAM/ATAF/CUC (NAC) TFs; MYC, bHLH subgroup IIIe TFs.

JA-Ile perception and signaling are implemented by SCF^COI1^-JAZ co-receptor complex. COI1, ASK2, CULLIN1, RBX1, and E2 are components of the SCF^COI1^ complex. The coding genes for these proteins showed almost no significant differential expression during the feeding process of the beet armyworm larvae (Table S13). JAZ proteins are the substrates of SCF^COI1^ and the main repressors of JA response. Eleven out of thirteen *Arabidopsis JAZs* (*JAZ1*–*JAZ13*) retained homologous genes in mustard (*JAZ4* and *JAZ11* were lost), and each contained three to six paralogs. It is worth noting that out of the 51 genes encoding these 11 JAZs, 44 genes were upregulated at least one time point after insect feeding, and the upregulation became more significant as feeding continued (Figure 8, Table S13). It is worth noting that all five genes encoding JAZ9 were significantly upregulated at four time points after insect attack (Figure 8, Table S13). Additional repression of JA signaling can take place by the Novel Interactors of JAZ (NINJA)/TOPLESS (TPL) via Histone Deacetylase 6 and 19 (*HDA6*, *HDA19*). However, the 14 genes encoding these four proteins showed no significant differences in expression before and after insect feeding (Table S13). The bHLH subgroup Ⅲd TFs, including bHLH17/Jasmonate-Associated MYC2-like TF (JAM1), bHLH13/JAM2, bHLH3/JAM3, and bHLH14, are another group of JA signaling repressors that antagonize the transcription activators (e.g., MYC2) by competitively binding to mutual target genes, thereby inhibiting the JA response. Apart from bHLH14 which was lost in mustard, there were 2, 1, and 1 mustard homologous genes encoding JAM1, 2, 3 that are significantly upregulated after insect feeding (Figure 8, Table S13).

For the JA signaling activator, as mentioned above, the master regulator of JA response, *MYC2*, and its two functional redundant genes, *MYC3* and *MYC4*, have 3, 2 and 2 mustard homologous genes upregulated after insect feeding, respectively. In addition, the direct targets of *MYC2*, *NAC019*, *NAC055* and *NAC072*, also have 2, 1 and 1 upregulated DEGs, respectively (Figure 8, Table S13). 

Apart from the aforementioned, there were no other noticeable JA signaling suppressors and activators that exhibited significant changes in expression due to insect attack. Interestingly, in both JA signaling repressors JAZs and JAMs, and activators MYCs and NACs, the number of up-DEGs peaked at 48 h of herbivory, followed by 72 h, 24 h, and 1 h (Table 1). In general, during the feeding process of beet armyworm larvae, the strength of anti-insect defense response was regulated by JA signaling repressors JAZs and JAMs, as well as the activators MYCs and NACs. Moreover, the game of the antagonistic relationship between activation and inhibition of anti-insect defense may be most intense at 48 to 72 h of insect feeding, as during this time, there was the largest number of differentially expressed JA signaling related regulators.

## 3. Discussion

The response of plants to highly complex and variable biotic stress conditions is predominately mediated by plant hormone signals [36] and secondary metabolites [37]. It has been fully demonstrated that the endogenous hormone JA and secondary metabolite glucosinolates play great roles in improving the resistance of plants to herbivorous insect stress in cruciferous plants [38,39]. Moreover, JA is the dominant positive regulator of multiplying GSL levels via JA signaling, while other hormones generally only modulate the basal GSL levels [40,41]. In this study, the changes in GSL profile and transcriptome analysis results of mustard after challenged with beet armyworm larvae once again showed that there was a causal relationship between the insect-induced JA pathway and GSL accumulation in cruciferous plants. Remarkably, we also revealed that JA-dependent responses do not only remain activated during insect feeding, but gradually enter a balance between activation and inhibition of anti-insect defense response, which is achieved by regulating the homeostasis of JA compounds and balancing the suppressors and activators of JA signaling at the transcriptional level.

### 3.1. The Accumulation of GSLs in Mustard Leaves after Herbivory Depends on the Upregulation of a Large Number of BjuGSL Genes

In the Brassicaceae family, the induced resistance against insects is partly dependent on GSLs (i.e., aliphatic and indolic GSLs), among which aliphatic GSLs have been proved to be effective against generalist herbivores [42,43]. Previous studies have demonstrated that the aliphatic GSL pathway is upregulated after feeding by *Spodoptera littoralis*, *Pieris brassicae* and *Pieris rapae* [43,44,45]. A high content of aliphatic GSLs, SIN and GIB, provided significant defenses against *Mamestra brassicae* in *B. oleracea* mature plants [46]. In addition, correlation coefficient analysis showed that the larval weight gain of the generalist pest (*Spodoptera litura*) was negatively correlated with SIN, GNA, and GBN in *B. juncea* [19]. It was reported that the high content of aliphatic SIN could decrease the number of generalist *M. brassicae* [47]. Several studies showed that more than 90% of GSLs were aliphatic GSLs in vegetable mustard, of which SIN was predominant [48,49]. A similar result was also found in this study. The total GSL and aliphatic GSL content was increased upon beet armyworm larvae infestation, with aliphatic GSLs accounting for 98% of the total GSLs (Figure 1A). The main GSL detected in mustard leaves was also SIN, accounting for more than 95% of the total GSLs (Figure 1B, Table S1), which once again indicates the importance of SIN in insect resistance. Mustard (AABB) is an allopolyploid species with an intricate evolutionary history, which have experienced many duplication events after interspecific hybridization between *B. rapa* (AA) and *B. nigra* (BB) [50,51,52,53]. A previous study from our group identified that SIN is also the dominant GSL in *B. nigra* leaves [34], which leads us to speculate that the specific GSL pattern dominated by SIN in mustard may mainly be caused by those *BjuGSLs* derived from the *B. juncea* B subgenome.

Transcriptome analysis showed that nearly half of the 209 *BjuGSLs* were differentially expressed in response to insects, and nearly 90% of DEGs were upregulated. GO and KEGG analysis also consistently revealed that the GSL biosynthesis pathway was significantly enriched by DEGs. Moreover, almost all steps from methionine to the final synthesis of SIN involve up-DEGs (Figure 4, Table S8). Among numerous up-DEGs, some *BjuGSLs* have attracted our attention and may contribute more to the accumulation of GSLs after herbivory. *BCAT4* controls the first step of methionine chain elongation pathway that leads to the biosynthesis of aliphatic GSL, and knockout of *BCAT4* significantly reduced the production of methionine-derived GSLs [54]. Transcriptome data showed that four paralogs of *BCAT4* were all induced by herbivory, and the expression of *BjuVB01G34920* was consistently upregulated within 72 h of insect feeding (Figure 4A, Table S8). Both *GGP1* and *SOT16* are involved in biosynthesis of core structure of GSLs, with *GGP1* reduces the accumulation of GSH-conjugate and increases the production of GSLs in conjunction with GSL biosynthesis genes [55,56]. Here, we found that all four paralogs of *SOT16*, as well as five of the six *GGP1* paralogs were up-DEGs, of which *BjuVB05G56430* (*SOT16*) and *BjuVB05G07180* (*GGP1*) were continuously upregulated and highly expressed during the 24 to 72 h of insect feeding. As the main constituent of aliphatic GSLs in vegetable mustard, the final synthesis step of SIN is catalyzed by an enzyme called AOP2. Previous findings indicated that the gene *AOP2* could feedforward the expression of genes involved in the JA signaling pathway to enhance the content of GSLs [57]. In our study, we found that four out of five *AOP2* paralogs were highly expressed and significantly induced by herbivory. In conclusion, the accumulation of GSLs (especially SIN) after insect feeding, is dependent on the upregulation of a large number of GSL biosynthesis genes, especially *BCAT4*, *GGP1*, *SOT16*, and *AOP2* mentioned above, which may contribute more and are worth further exploration.

Interestingly, when considering the upstream direct regulatory factors of *BjuGSLs*, it was found that most MYBs were not significantly upregulated, while MYCs that can form complexes with MYBs and regulate GSL synthesis were upregulated after herbivory (Figure 5). It has been reported that upon herbivory and activation of the JA pathway, JAZ proteins are degraded by the SCF^COI1^ complex, allowing MYCs to be released, thereby interacting with MYBs and activating the transcription of GSL biosynthesis genes [25]. Given the significant upregulation of JA biosynthesis-related genes and activation of the JA pathway (discussed below), we believe that the induction of *BjuGSLs* is partly due to the upregulation of MYCs, but more importantly, it may be due to JA signaling triggering a stronger MYC–MYB association. In addition, the regulation of *BjuMYB29* (*BjuVA10G29940*) on *BjuGSLs* under insect attack is also worth further evaluation, as we recently found that exogenous JA can significantly induce the expression of this gene (data not shown).

### 3.2. The JA-Dependent Response Induced by Herbivory Is Fine-Tuned by Regulating the Homeostasis of JA Compounds and the Activation of JA Signaling Regulators

The defense response of plants to insects is regulated by molecular signals, of which the most important is JA [58,59]. The rapid accumulation of JA in the early response process of herbivory depends on the conversion of precursor substance alpha-linolenic acid [60,61,62,63]. In this study, a total of 32 DEGs involved in the alpha-linolenic acid metabolism and the JA biosynthesis were all upregulated on at least one time point after herbivory (Figure 3C and Figure 6, Table S10), implying the continuous synthesis of JA. Studies showed that LOXs (including LOX2, LOX3, LOX4 and LOX6) are wound-induced antioxidative enzymes that play a critical role in defense response to biotic stress (i.e., insects, pests, and pathogenic attacks) [64,65,66]. Here, we found that most *LOXs* were induced from 24 h after herbivory, and the expression of three *LOX2* genes were the highest, which rapidly increased from 1 h to 72 h after herbivory (Figure 6, Table S10). High transcription of *AOS* genes and enhanced activity of AOC are usually observed in wounded leaves [65]. *AOS* is involved in the biosynthesis of herbivore-induced JA and plays an important role in rice’s defense against herbivory [67]. OPDA produced in chloroplasts is transported into peroxisomes and then reduced by OPR3, which can regulate a variety of signal functions and enhance plant resistance to insects [68,69]. In the present study, two *AOS* genes, six *AOC* genes, and two *OPR3* genes were upregulated after herbivory, and all reached their highest expression levels at 72 h (Figure 6, Table S10). The upregulation of these JA biosynthesis-related genes allows for the continuous accumulation of JA after herbivory, thereby ensuring the response of JA to resist insect feeding.

The growth and defense of plants have always been two major themes in plant research. A study in a mutant with mutations in five repressors of JA signaling and the photoreceptor phyB showed that constitutive defense and growth can occur simultaneously, suggesting that the growth–defense tradeoff was uncoupled [70]. The JAZ-MYC transcriptional module constructed by Major et al. [29] further unravels the complexities of JAZ–TF interactions, demonstrating that MYC TFs act as master regulators on the JAZ-repressible transcriptional hierarchy that controls the growth–defense balance. Therefore, it is worth discussing whether plants also regulate anti-insect defense responses through JA-related processes during the continuous attack of insects to avoid over-stunted growth due to excess defense response. 

The processes related to JA can be roughly summarized as the JA biosynthesis, JA metabolism, and JA signaling. We have discussed above that insect feeding induces significant upregulation of a large number of JA biosynthesis genes, ensuring the activity of the JA synthesis pathway. Many genes related to JA metabolism and JA signaling were also differentially expressed after herbivory. It is known that JA can be converted into active and inactive compounds, or partially active compounds, through various metabolic pathways [66,71,72]. JA-Ile, as a ligand mediating the formation of SCF^COI1^-JAZ co-receptor complex, is considered the most biologically active JA compound, and plays an important regulatory role in all JA-dependent processes [73,74,75,76]. JAR1 is the most important but may not be the only JA-conjugating enzyme that converts JA into JA-Ile [77,78]. In this study, we found that three of the six *JAR1* homologous genes were induced by insect feeding. Interestingly, several amido-hydrolases, including *IAR3* and *ILL6*, which are responsible for cleaving JA-Ile into JA [72], are also activated. In addition, several enzymes that catalyze the conversion of other JA compounds have also found to be induced by herbivory, including *JMT*, *JOX1*/*2*/*3*/*4*, *ST2A*, *CYP94B3* and *CYP94C1* (Figure 7). It has been reported that the dynamics of JA-Ile homeostasis differ between leaf injury, developing flowers, and *Botrytis cinerea*-infected leaves [79]. Here, the induction of a large number of JA metabolism-related enzymes also implies the complex sustainment of JA-Ile homeostasis during the feeding process of the beet armyworm larvae.

The discovery and identification of JAZ proteins, the substrate of SCF^COI1^ complex, are one of the important milestones in JA signaling [74,80]. Overexpression of *JAZ4*, *JAZ7*, *JAZ8*, *JAZ9*, *JAZ13*, alternative splice variants or truncated forms of JAZs can inhibit defenses against the herbivore *Spodoptera exigua* or the pathogen, and single or multiple mutants of some JAZ genes exhibit susceptibility to *Pseudomonas syringae*. It has been reported that with the accumulation of JAZs, the JA responses is attenuated [39]. Here, we found that as many as 44 mustard homologous genes encoding 11 JAZ proteins were upregulated by insect feeding (Figure 8), which means that the JA response may be partially inhibited with the increase in *JAZs* level. In addition to *JAZs*, the bHLH subgroup Ⅲd TFs, including JAM1, JAM2, JAM3 and bHLH14, also function redundantly as transcriptional repressors and inhibit various JA responses, including defense against insect attack [81,82,83,84]. A total of four mustard homologous genes encoding JAM1/2/3 were induced by herbivory (Figure 8). For JA signaling activators, there are seven and four genes encoding MYCs and NACs that were upregulated after insect feeding, respectively.

In summary, during the continuous feeding process of beet armyworm larvae, a large number of genes involved in JA-related processes are induced, including JA biosynthesis genes (*LOXs*, *AOSs*, *AOCs*, *OPR3*, and *OPCL1*), catalytic enzyme coding genes for JA compound conversion (*JAR1*, *IAR3*, *ILL6*, *JMT*, *JOXs*, *LOXs*, *ST2A*, and *CYP94B3/C1*), and JA signaling repressors (*JAZs* and *JAMs*) and activators (*MYCs* and *NACs*). As discussed above, the upregulation of some genes has a promotional effect on the JA response, while others have an attenuating effect, suggesting that the JA response regulation during insect feeding is very complex and fine-tuned by regulating the homeostasis of JA compounds and the activation of JA signal regulatory factors. Future research is needed to determine the roles of these genes in this delicate regulatory network, which could provide insights for achieving high insect resistance through fine-tuning genes without sacrificing Brassica yield in the future.

## 4. Materials and Methods

### 4.1. Plant and Insect Materials

The leaf mustard genotype used in this experiment was *B. juncea* cv. Xuelihong (‘Zhenong No 1′, a five-generation inbred line in our laboratory), which is one of the most widely cultivated leaf mustards in China. Mustard plants were planted in an artificial climate chamber under 25 °C/18 °C, 14/10 h day/night, and 60–70% relative humidity. Plants with nine fully developed leaves were selected for the experiment. The beet armyworm larvae were reared artificially in an artificial climate chamber at 28 °C with a 12:12 h photoperiod and 60–70% relative humidity. For beet armyworm larvae treatments, ten third-stage larvae starved for 5 h were placed on five leaves of seedlings. The control group comprised similar plants maintained under the same conditions without exposure to the larvae of beet armyworm. Insect-damaged leaves from three plants were harvested after the onset of 1, 24, 48 and 72 h of herbivory. Three biological replicates were used in each experiment. All the materials at each time points were immediately frozen in liquid nitrogen and then stored at −80 °C.

### 4.2. Glucosinolate Analysis

The analysis of GSLs was performed by high-performance liquid chromatography (HPLC). The GSL extraction and analysis procedures were performed as previously described with a slight modification [34]. A total of 0.25 g of the sample powder was boiled with 10 mL of 70% methanol and the supernatant was applied to a DEAE-Sephadex A-25 column. Glucotropaeolin (Applichem) was used as an internal standard for HPLC analysis. The resultant desulfoglucosinolates were eluted with ultrapure water and stored at 4 °C until analysis. An Agilent 1200 HPLC system equipped with a C-18 reversed phase column (250 × 4 µm, 5 µm, Bischoff, Leonberg, Germany) was used with a mobile phase of ultrapure water (solvent A) and acetonitrile (solvent B). The analytical conditions were: flow rate at 1 mL min^−1^ (injection volume 20 µL), detection wavelength at 229 nm.

### 4.3. Statistical Analysis

SPSS 22.0 software was used for GSL concentration data analysis. Statistical analysis was performed by one-way analysis of variance (ANOVA) followed by Fisher’s least significant difference (LSD) test. The data of GSL concentrations were shown as mean ± standard deviation (SD), and the values were considered significant if *p* < 0.05. Graphs were generated using the GraphPad Prism 8.0 analysis software.

### 4.4. RNA Extraction, Library Construction, Sequencing and Assembly

Total RNA was isolated from the mustard leaves using Trizol (Invitrogen, Waltham, MA, USA) according to the manufacturer’s instructions. The sequencing and assembly were performed as recently described by the Biomarker Biotechnology Corporation (Beijing, China) using the Illumina HiSeq^TM^ 2500 platform [34]. The genome sequences of the *B. juncea* genome (Braju_tum_V2.0) were downloaded from the Brassica database (http://brassicadb.cn/#/Download/, accessed on 18 January 2022).

### 4.5. Differential Expression Genes (DEGs)Analysis

Differential expression analysis of different groups was performed using the DESeq2 R package (1.20.0) [85]. The resulting *p*-values were adjusted using Benjamini and Hochberg’s approach, the false discovery rate padj ≤0.05, and |log2(foldchange)| ≤ 1 were set as the threshold for significantly differential expression. A Venn diagram was generated using an online tool Venny 2.1.0 (Oliveros, J.C., 2007–2015, https://bioinfogp.cnb.csic.es/tools/venny/index.html, accessed on 20 April 2023). The Multiexperiment Viewer software (v4.9) was employed to exhibit the expression profiles of DEGs by clustering, and cluster analysis of the DEGs was based on the *K*-means method [86]. Heatmaps of DEGs were generated using the TBtools software [87].

### 4.6. Functional Analysis of DEGs

Gene ontology (GO) enrichment analysis of DEGs was performed using the ClueGO plugin of Cytoscape, and a two-sided hypergeometric test using the Bonferroni-step-down multiple-hypothesis-testing correction [88,89]. In addition, the Kyoto Encyclopedia of Genes and Genomes (KEGG) was used to analyze the metabolic pathways involved by DEGs. ClusterProfiler R package (3.8.1) was utilized to test the statistical enrichment of DEGs in KEGG pathways [90], and the pathways with *p*-value ≤ 0.05 were considered significantly enriched. Protein–protein interaction (PPI) network was constructed based on the STRING database (https://string-db.org/, accessed on 11 May 2023), and the images were examined using the NetworkAnalyst platform [91,92]. Using a confidence score higher than 900 and experimental evidence required as parameters and the minimum connected network option, the significant genes were mapped to the corresponding molecular interaction database.

## 5. Conclusions

This study explored the GSL profile and the molecular defense mechanism of *B. juncea* against the generalist insect, beet armyworm larvae. The results showed that the accumulation of total GSLs mainly depends on the increase in aliphatic GSL SIN. Further transcriptome analysis revealed that insect attack may induce JA biosynthesis and then promote GSL accumulation to enhance insect resistance. Furthermore, many genes related to JA catabolism and JA signaling were also upregulated revealing a complex regulatory mechanism that involves regulating the homeostasis of JA compounds and balancing the suppressors and activators of JA signaling at the transcriptional level during insect chewing, leading plants to enter the activation-inhibition tradeoff state of anti-insect defense responses. This study provides data to guide further research into the mechanism of Brassica plants responding to insect attacks, as well as ideas for improving insect resistance with little or no yield loss.

## Figures and Tables

**Table 1:** The number of up-DEGs in the JA signaling repressors JAZs and JAMs, and the activators MYCs and NACs.

	1 h/CK	24 h/CK	48 h/CK	72 h/CK
JAZs	7	22	44	41
JAMs	1	3	4	3
MYCs	2	1	4	4
NACs	0	1	4	2

## Data Availability

Data are presented in the manuscript and in the Supplementary Materials.

## Supplementary Materials

The following supporting information can be downloaded at: https://www.mdpi.com/article/10.3390/ijms242316690/s1.
