# Supplementary material for: Transcriptome Dynamics of Brassica juncea Leaves in Response to Omnivorous Beet Armyworm (Spodoptera exigua, Hübner)
Source: Int J Mol Sci. 2023 Nov 24;24(23):16690. doi: 10.3390/ijms242316690 (PMC10706706; doi:10.3390/ijms242316690)
Supplement: Supplementary file 1 [file ijms-24-16690-s001.zip › Figure S1-S3.pdf]

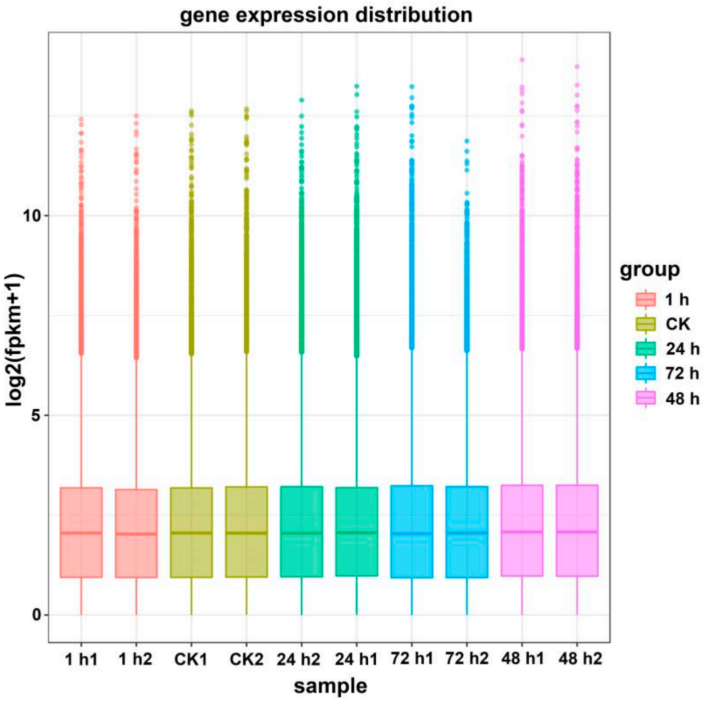

Figure S1. Box diagram of the gene expression distribution of each sample after feeding on mustard leaves by beet armyworm larvae. The X-axis represents sample name. The Y-axis represents log2 (fpkm + 1). Fpkm (expected number of Fragments Per Kilobase of transcript sequence per Millions base pairs sequenced) refers to the number of fragments per Kilobase length from a gene in per million fragments.

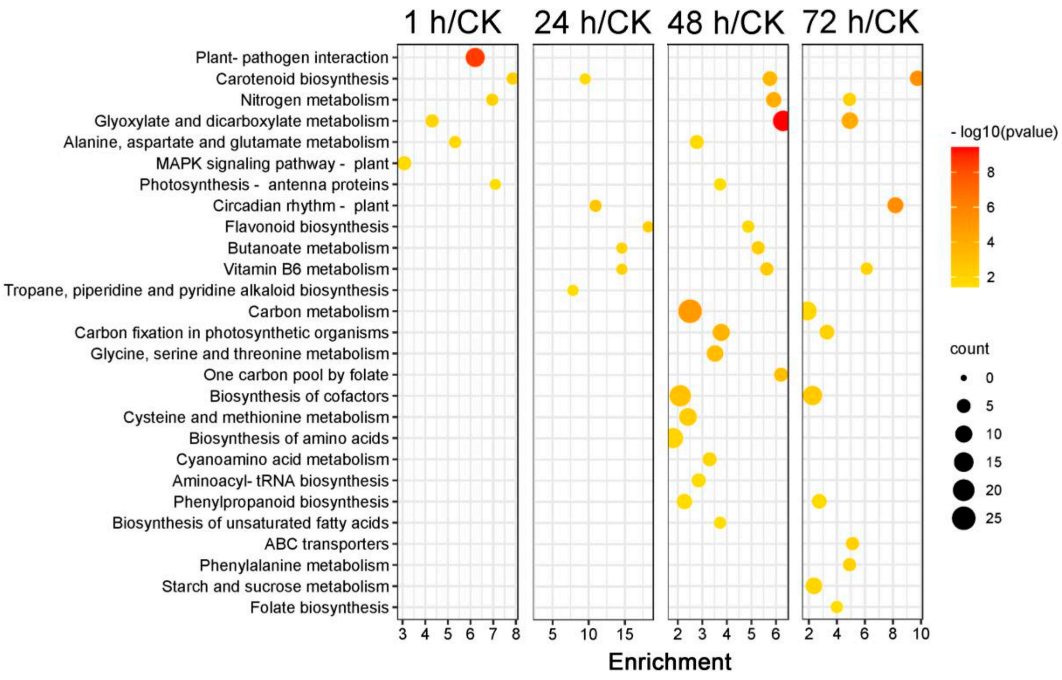

Figure S2. KEGG classification of the down-regulated DEGs. The X-axis represents the enrichment, which is the ratio of the number of differentially expressed genes to the total number of genes in a certain pathway. The color and size of the dots represent the range of the -log10(pvalue) and the number of DEGs mapped to the indicated pathways, respectively.

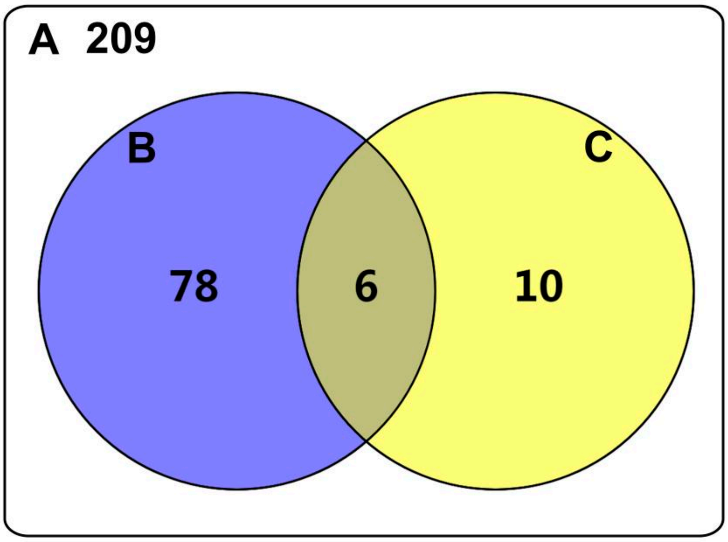

Figure S3. The number of DEGs involved in GSL biosynthesis and sulfur assimilation which have a different expression pattern after beet armyworm larvae chewing in *B. juncea*. (**A**) The total number of genes related to GSL biosynthesis and sulfur assimilation in *B. juncea*. (**B**) The DEGs which have been up-regulated at least at one time point after beet armyworm chewing in *B. juncea*. (**C**) The DEGs which have been down-regulated at least at one time point after beet armyworm chewing in *B. juncea*.
