# Supplementary material for: Transcriptome Dynamics of Brassica juncea Leaves in Response to Omnivorous Beet Armyworm (Spodoptera exigua, Hübner)
Source: Int J Mol Sci. 2023 Nov 24;24(23):16690. doi: 10.3390/ijms242316690 (PMC10706706; doi:10.3390/ijms242316690)
Supplement: Supplementary file 1 [file ijms-24-16690-s001.zip › Table S1-S2, S6-S7.pdf]

Table S1. Glucosinolate content and constituent in the leaves of *Brassica juncea* after beet armyworm larvae chewing.

	Progoitrin	Sinigrin	Glucoraphanin	Gluconapin	4-OH-Glucobr assicin	Glucobrassicin	4-Methoxy-Glu cobrassicin	1-Methoxy-Glu cobrassicin
CK	0.125±0.015b[1]	49.474±9.741bc	0.198±0.013a	0.228±0.042bc	0.334±0.032b	0.157±0.004d	0.463±0.044ab	0.078±0.003a
1 h	0.097±0.011b	41.893±2.927c	0.175±0.001ad	0.211±0.051c	0.397±0.036ab	0.175±0.011d	0.333±0.059ab	0.074±0.004a
24 h	0.190±0.002a	51.360±2.186bc	0.180±0.020ab	0.248±0.002bc	0.376±0.004ab	0.218±0.008c	0.292±0.052b	0.076±0.021a
48 h	0.170±0.008a	63.648±6.774ab	0.177±0.013ac	0.315±0.033ab	0.429±0.049a	0.326±0.019b	0.417±0.034ab	0.090±0.005a
72 h	0.179±0.024a	70.891±0.141a	0.148±0.007bcd	0.376±0.020a	0.358±0.026ab	0.560±0.024a	0.495±0.124a	0.097±0.020a

[1]Data are means ± SD. Data unit is μM/g DW. DW: dry weight. Means followed by different letters in each row are significantly different ($P < 0.05$).

Table S2. Overview of the mustard transcriptome sequencing analysis and assembly.

Samples	Clean reads	Clean bases	GC Content	%≥Q30	total_map	unique_map
CK1	43,317,162	6.50 G	47.61%	90.95%	39,530,337(91.26%)	37,738,395(87.12%)
CK2	41,852,788	6.28 G	47.68%	91.39%	38,198,174(91.27%)	36,414,338(87.01%)
1 h1	44,926,432	6.74 G	47.48%	90.56%	40,766,116(90.74%)	38,940,895(86.68%)
1 h2	42,881,498	6.43 G	47.20%	90.99%	39,100,055(91.18%)	37,412,924(87.25%)
24 h1	45,196,916	6.78 G	47.88%	91.17%	41,479,437(91.77%)	39,522,455(87.45%)
24 h2	38,645,702	5.80 G	47.79%	90.82%	35,016,474(90.61%)	33,247,082(86.03%)
48 h1	47,150,356	7.07 G	47.91%	91.19%	43,264,370(91.76%)	41,477,546(87.97%)
48 h2	46,659,404	7.00 G	47.94%	90.67%	42,214,323(90.47%)	40,126,611(86.0%)
72 h1	45,956,978	6.89 G	48.26%	91.44%	41,993,518(91.38%)	39,861,785(86.74%)
72 h2	45,807,882	6.87 G	47.01%	91.26%	41,361,695(90.29%)	39,245,744(85.67%)

Note: Clean reads: the number of reads after raw data filtering; Clean bases: the number of bases after raw data filtering(clean base = clean reads * 150bp); GC content: the percentage of GC-content in clean data; %≥Q30 : Q-score of clean data ≥30; Total_map: the number of reads mapped to the reference genome and the percentage in clean reads; Unique_map: the number of reads mapped to the unique location of the reference genome and the percentage in clean reads.

Table S6. KEGG enrichment analysis of up-regulated DEGs was compared among comparison pairs.

Pathway	1 h/CK_up	24 h/CK_up	48 h/CK_up	72 h/CK_up
Ribosome	1.30576E-07	-	-	-
Protein processing in endoplasmic reticulum	5.74261E-05	-	-	-
Ribosome biogenesis in eukaryotes	0.001118647	-	-	-
Glucosinolate biosynthesis	0.016075141	4.1023E-07	0.008730686	5.22626E-05
Sulfur metabolism	0.020575896	-	-	-
alpha-Linolenic acid metabolism	-	5.88148E-07	6.88526E-07	4.04523E-06
2-Oxocarboxylic acid metabolism	-	6.69894E-05	0.018802359	0.002335593
Tryptophan metabolism	-	0.008622389	0.017087506	0.01123207
Monobactam biosynthesis	-	0.021032176	0.019560299	-
Valine, leucine and isoleucine biosynthesis	-	0.032251211	-	-
Photosynthesis - antenna proteins	-	-	9.05652E-06	-
Plant hormone signal transduction	-	-	0.000374367	-
Phenylpropanoid biosynthesis	-	-	0.004623033	-
Cysteine and methionine metabolism	-	-	0.007895114	0.000737719
Tyrosine metabolism	-	-	0.013390588	0.006475569
Tropane, piperidine and pyridine alkaloid biosynthesis	-	-	0.013390588	0.006475569
Zeatin biosynthesis	-	-	0.03396591	0.002644423
Nitrogen metabolism	-	-	0.03396591	-
Phenylalanine metabolism	-	-	0.038618567	-
Phenylalanine, tyrosine and tryptophan biosynthesis	-	-	-	0.000257525
Biosynthesis of nucleotide sugars	-	-	-	0.00577754
Glycine, serine and threonine metabolism	-	-	-	0.008316362
Biosynthesis of various plant secondary metabolites	-	-	-	0.008752632
Glutathione metabolism	-	-	-	0.019279552
Amino sugar and nucleotide sugar metabolism	-	-	-	0.02745209
Isoquinoline alkaloid biosynthesis	-	-	-	0.030102974

Table S6. *Cont.*

Pathway	1 h/CK_up	24 h/CK_up	48 h/CK_up	72 h/CK_up
Biosynthesis of amino acids	-	-	-	0.034518718
Ascorbate and aldarate metabolism	-	-	-	0.040972603
Other glycan degradation	-	-	-	0.047364498

The value in the table is pvalue<0.05.

Table S7. KEGG enrichment analysis of down-regulated DEGs was compared among comparison pairs.

pathway	1 h/CK_down	24 h/CK_down	48 h/CK_down	72 h/CK_down
Plant-pathogen interaction	2.19296E-08	-	-	-
Carotenoid biosynthesis	0.00606882	0.018160529	0.00040858	3.71884E-06
Nitrogen metabolism	0.008601466	-	0.000109903	0.007994668
Glyoxylate and dicarboxylate metabolism	0.013210045	-	3.63488E-10	6.37487E-05
Alanine, aspartate and glutamate metabolism	0.018029031	-	0.03174976	-
MAPK signaling pathway - plant	0.022166105	-	-	-
Photosynthesis - antenna proteins	0.031379025	-	0.043502909	-
Circadian rhythm - plant	-	0.00238042	-	3.22446E-06
Flavonoid biosynthesis	-	0.005043319	0.02101101	-
Butanoate metabolism	-	0.007887468	0.005646499	-
Vitamin B6 metabolism	-	0.007887468	0.004395014	0.011546659
Tropane, piperidine and pyridine alkaloid biosynthesis	-	0.026375777	-	-
Carbon metabolism	-	-	1.25883E-05	0.018531519

Table S7. *Cont.*

pathway	1 h/CK_down	24 h/CK_down	48 h/CK_down	72 h/CK_down
Carbon fixation in photosynthetic organisms	-	-	0.000232675	0.009069978
Glycine, serine and threonine metabolism	-	-	0.000815045	-
One carbon pool by folate	-	-	0.000873027	-
Biosynthesis of cofactors	-	-	0.001420707	0.003090147
Cysteine and methionine metabolism	-	-	0.005118296	-
Biosynthesis of amino acids	-	-	0.014275364	-
Cyanoamino acid metabolism	-	-	0.015985815	-
Aminoacyl-tRNA biosynthesis	-	-	0.028632371	-
Phenylpropanoid biosynthesis	-	-	0.032426124	0.020883098
Biosynthesis of unsaturated fatty acids	-	-	0.043502909	-
ABC transporters	-	-	-	0.006885969
Phenylalanine metabolism	-	-	-	0.007994668
Starch and sucrose metabolism	-	-	-	0.013142711
Folate biosynthesis	-	-	-	0.037219264

The value in the table is pvalue<0.05.
